# Morgagni hernia associated with ipsilateral lateral chest wall defect: a case report of transdiaphragmatic intercostal hernia in an adult

**DOI:** 10.1097/MS9.0000000000004610

**Published:** 2026-01-06

**Authors:** Minami Watanabe, Richard S. Chang

**Affiliations:** a Department of General Surgery, Lehigh Valley Health Network, Allentown, PA, USA; b Department of Cardiothoracic Surgery, Lehigh Valley Health Network, Allentown, PA, USA

**Keywords:** chest wall defect, congenital diaphragmatic hernia, Morgagni hernia, transdiaphragmatic intercostal hernia

## Abstract

**Introduction and Importance:**

Transdiaphragmatic intercostal hernias (TDIHs) are rare and typically posttraumatic. The coexistence of a TDIH with congenital anomalies, such as a Morgagni hernia (MH) and chest wall defect, is exceptionally uncommon. This report details a unique case of MH associated with an ipsilateral intercostal hernia in an adult, underscoring key diagnostic and surgical challenges.

**Case Presentation:**

A 71-year-old man with multiple comorbidities presented with acute abdominal pain and a right-sided chest wall bulge. Computed tomography revealed a large right lateral diaphragmatic and intercostal hernia between the right 9th and 10th ribs, with bowel loops protruding through the intercostal defect. The symptoms spontaneously improved, and repeat imaging showed a reduction of the hernia content. Thoracotomy revealed an omental herniation through an anteromedial diaphragmatic defect (MH) and a congenital chest wall defect with stretched intercostal muscles. The MH was primarily repaired using nonabsorbable sutures, and rib approximation was performed with absorbable sutures to address the chest wall defect.

**Clinical Discussion:**

This case is remarkable for the association of two congenital defects: an MH and a TDIH. Unlike typical posttraumatic TDIHs, the intercostal defect in this case stemmed from congenital chest wall maldevelopment. Preoperative diagnosis was challenging, with the MH initially missed on imaging. This emphasizes the importance of considering congenital variants in atypical hernias.

**Conclusion:**

To our knowledge, this is the first documented case of a TDIH occurring in conjunction with an MH, both likely congenital. Prompt recognition and tailored interventions are crucial for avoiding missed diagnoses and complications.

## Introduction

Intercostal hernia is an uncommon condition characterized by disruption or weakness of the thoracoabdominal wall musculature, resulting in protrusion of thoracoabdominal viscera through a defect between two consecutive ribs. When associated with diaphragmatic defects, it is referred to as a transdiaphragmatic intercostal hernia (TDIH). TDIHs are exceedingly rare and are typically associated with trauma[1]. Given its rarity, establishing an early diagnosis and selecting the most appropriate treatment remain challenging. The coexistence of TDIH with congenital anomalies – such as a Morgagni hernia (MH) and a chest wall defect – is exceptionally uncommon. We present a rare confluence of an MH and ipsilateral intercostal hernia in a 71-year-old man, highlighting the diagnostic and surgical challenges involved in managing such complex cases. This case report has been prepared in accordance with the Surgical CAse REport (SCARE) criteria[2].

## Case presentation

### Presentation and history

A 71-year-old man with a complex medical history, including chronic kidney disease, type 2 diabetes mellitus, bariatric sleeve gastrectomy, coronary artery disease, and aortic root aneurysm status after coronary artery bypass graft with bioprosthetic aortic root replacement, presented with acute-onset worsening abdominal pain. The patient reported a longstanding right-sided chest and abdominal wall bulge, first noted approximately 5 years prior, with intermittent bulging and discomfort exacerbated by physical exertion. The patient denies any history of prior chest trauma, thoracic surgery, or rib fractures.

### Physical examination and diagnosis

**Figure 1.**
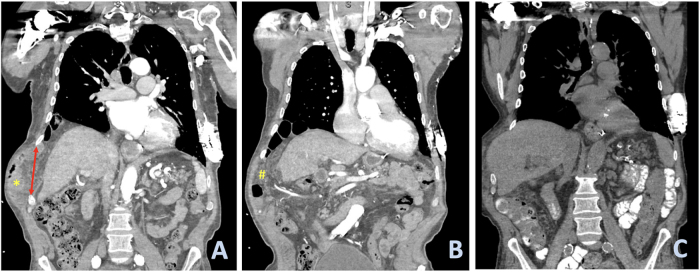
Pre- and postreduction CT scans. Herniation of bowel (* in image A) and omentum (# in B) into the right hemithorax, with associated bulging of the right thoracic wall and widening of the 10th intercostal space (double arrow in A). After spontaneous reduction of bowel and omentum, herniation of the liver into the right hemithorax through the widened 10th intercostal space is visible (C).

**Figure 2.**
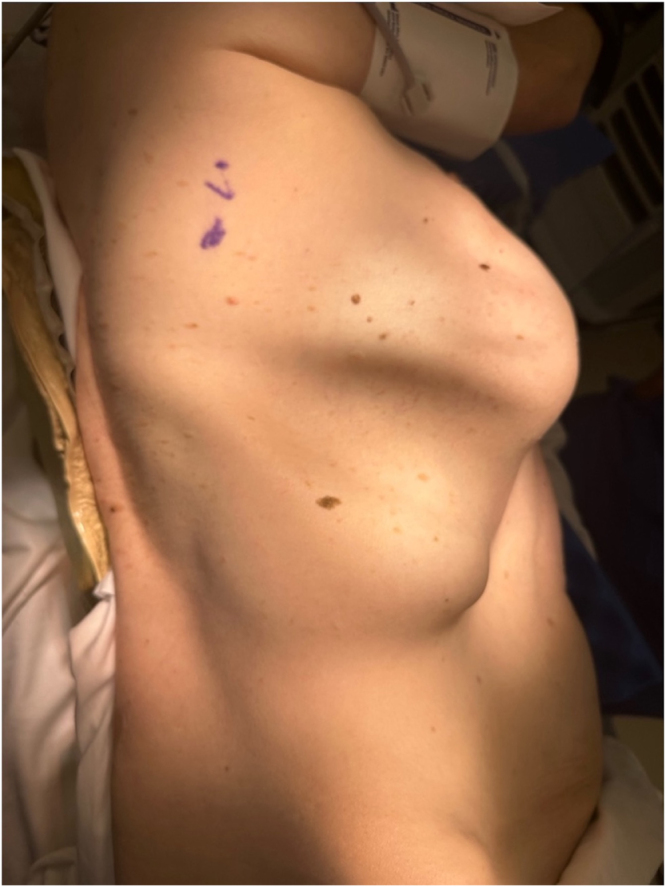
Intraoperative view of the right-sided chest wall defect, characterized by laxity and maldevelopment of the intercostal muscles.

Physical examination revealed bulging of the right lateral chest wall with partially reducible hernias. Computed tomography (CT) scan obtained upon admission revealed a large right lateral diaphragmatic hernia and an intercostal hernia between the right 9th and 10th ribs, with bowel loops protruding through the intercostal defect (Fig. 1). Notably, the patient’s symptoms spontaneously improved within 24 h. A repeat CT scan showed an interval reduction in the hernia contents (Fig. 2).

### Operative management and findings

**Figure 3.**
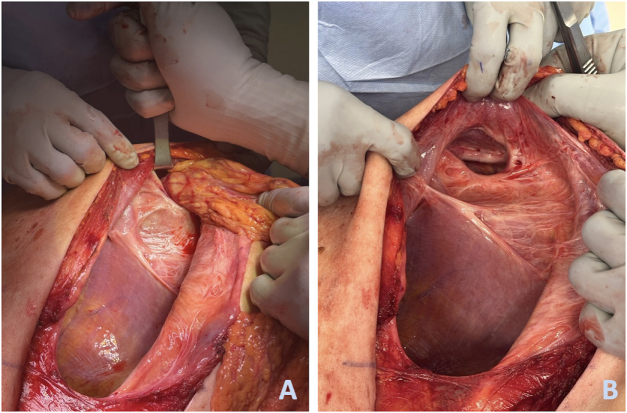
Intraoperative images of the Morgagni hernia. Omentum herniating through the defect (A) and the defect after reduction (B).

The patient was transferred to the operating room the following day. Upon positioning in the left lateral decubitus position (right side up), a large defect was noted between the 9th and 10th ribs (Fig. 3). A right-sided exploratory thoracotomy was performed through the 10th rib space defect. Stretched and maldeveloped intercostal muscles with partial agenesis were noted during thoracotomy dissection. Thoracotomy revealed omental herniation through a defect in the anteromedial aspect of the right diaphragm, consistent with MH. Bowel involvement was not observed. Additionally, a congenital right-sided chest wall defect was noted, which was attributed to hypermobility, stretching, and thinning of the intercostal muscles in the 10th rib space. The omentum was reduced without major issues. The MH orifice measured approximately 4 cm at its maximum diameter and was repaired primarily with nonabsorbable running sutures under no tension. The chest wall defect was reconstructed by rib approximation, mobilizing the 9th and 10th ribs and reinforcing the area with absorbable sutures. A 28-French chest tube was placed in the right thoracic cavity and the intercostal muscles, subcutaneous tissue, and skin were closed in layers.

### Postoperative course and follow-up

The postoperative course was uneventful. The chest tube was removed on the fourth postoperative day, and the patient was discharged the following day in a stable condition. At eight months postoperatively, surveillance chest CT demonstrated recurrence of the intercostal hernia without evidence of MH recurrence. The patient reported intermittent discomfort in the right lateral thoracoabdominal region and is currently not planning to pursue further operative intervention.

## Discussions

MH is a rare congenital diaphragmatic hernia characterized by a defect in the anterior part of the diaphragm, accounting for approximately 2–4% of all diaphragmatic hernias[3]. The majority (90%) of cases occur on the right side and are more commonly observed in females and obese individuals. Although typically diagnosed during childhood, approximately 10–30% of MHs remain asymptomatic and are discovered incidentally on imaging in adulthood[4]. In rare cases, acute abdominal or thoracic symptoms, often secondary to bowel obstruction or strangulation, may prompt further investigation and lead to diagnosis^[5,6]^. The pathophysiology of this condition lies in failure of fusion between the diaphragm and costal arches during embryogenesis. Although MHs are congenital, their manifestation in adulthood is believed to be precipitated by prolonged or sudden increases in intra-abdominal pressure. The contributing factors include obesity, pregnancy, chronic constipation, trauma, and persistent coughing[4].

**HIGHLIGHTS**

- This is the first documented case of a transdiaphragmatic intercostal hernia occurring concurrently with a Morgagni hernia in an adult, both likely of congenital origin.
- The intercostal defect was attributed to maldevelopment of the chest wall musculature, rather than the more typical post-traumatic etiology.
- Preoperative imaging failed to detect the Morgagni hernia, underscoring diagnostic limitations and the need for high clinical suspicion in atypical presentations.
- A thoracic surgical approach allowed for successful primary repair without mesh, and rib approximation was performed to minimize risk of recurrence.
- This case highlights the clinical importance of individualized surgical planning in rare hernia variants, particularly when standard imaging is inconclusive.

Intercostal hernias are categorized into three distinct types: (1) abdominal intercostal hernia, where intra-abdominal organs protrude through the intercostal space; (2) pulmonary intercostal hernia, in which the lung tissue herniates through the chest wall; and (3) TDIH, in which both diaphragm and intercostal muscles are disrupted[7]. The etiology of the vast majority of intercostal hernias is posttraumatic. Traumatic tears of the diaphragm often involve the posterior aspect of the diaphragm due to disruption of the posterolateral attachments and the weaker points of the diaphragm, and occur more frequently on the left side due to the protection of the right lobe of the liver to the right hemidiaphragm. According to a 2024 review by Ho *et al*, fewer than 50 cases of traumatic TDIH have been reported since the first documented case in 1946[8]. Nontraumatic causes are even rarer and include iatrogenic (e.g., postoperative), spontaneous (e.g., coughing), infectious, congenital, and neoplastic etiologies^[8,9]^.

In this case, two distinctive features make this noteworthy: (1) Associated diaphragmatic hernia was congenital MH, not disruption or tear of the diaphragm, (2) The intercostal defect was attributed to a chest wall abnormality, likely of congenital origin, as suggested by the stretched, maldeveloped intercostal musculature in the absence of prior trauma, with no distinct hernial sac or hernia defect. To the best of our knowledge, this is the first reported case of TDIH occurring in conjunction with a congenital MH rather than with an acquired diaphragmatic tear or disruption.

Diagnosing MH and/or TDIH can be challenging, requiring a thorough history and comprehensive physical examination because of the wide variety and nonspecific nature of the presenting symptoms. The utility of diagnostic modalities is highly dependent on a patient’s clinical presentation. Notably, in a review by Katsaros *et al*, 28% of MHs were undiagnosed preoperatively[4]. Nonetheless, CT remains the most sensitive and specific modality for definitive diagnosis of both MHs and TDIHs. This case highlights the need for increased awareness of rare congenital variants in patients with complex chest wall hernias because misdiagnosis or delayed diagnosis may result in inappropriate surgical planning or missed opportunities for optimal intervention. In our case, preoperative diagnosis was challenging, and the MH was initially overlooked on imaging because the anteromedial diaphragmatic defect was not clearly visualized. This difficulty likely stemmed from the limitations of CT imaging in visualizing diaphragmatic defects, especially when obscured by orientation or adjacent structures. Therefore, maintaining a high index of suspicion is essential, particularly in patients with atypical thoracoabdominal symptoms or inconclusive imaging findings. In such cases, a low threshold for diagnostic thoracoscopic or laparoscopic exploration may be warranted to ensure timely and accurate diagnosis.

Owing to its rarity, the surgical approach to both MH and TDIH remains variable with no universally accepted guidelines[10]. Several reports have been published on both traumatic and nontraumatic/spontaneous TDIH and its management, with significant variations in the surgical techniques[8]. In previously reported cases, surgical repair of TDIHs has been performed using approaches, including abdominal, thoracic, or thoracoabdominal approaches^[9,11,12]^. Reported approaches for hernia repair include primary suture repair with or without mesh reinforcement, rib plating if concurrent rib fracture occurs, or combinations thereof[7]. To date, four cases have been reported in which the etiology was specifically documented as nontraumatic, mostly due to coughing or sternutation. In all four cases, the diaphragmatic defect or hernia was repaired primarily without mesh^[9,11–13]^.

In our case, a thoracic approach was selected, given the predominant lateral chest wall involvement and reduced hernia contents on repeat imaging. Mesh was not utilized because the MH defect could be approximated without tension. Some studies recommend against rib approximation to avoid chronic pain secondary to intercostal nerve injuries. However, we elected to perform rib approximation to minimize the risk of future complications, such as lung herniation or recurrence.

Despite this, the patient subsequently developed a recurrent intercostal hernia, while the MH repair remained intact without recurrence on follow-up imaging, underscoring the differing reinforcement requirements between the two defects. This outcome suggests that, although primary repair without mesh may be sufficient for small diaphragmatic defects, reinforcement of the intercostal component should be considered to minimize the risk of recurrence, particularly when muscular hypoplasia or chest wall weakness is observed intraoperatively. The patient was advised to gradually resume physical activity postoperatively; however, it remains uncertain whether specific activity restrictions or lifestyle modifications might have altered the postoperative course. Close follow-up with routine imaging remains crucial given the potential risk of recurrence.

Although the intercostal musculature appeared maldeveloped intraoperatively, histopathologic confirmation was not obtained, representing a limitation in definitively establishing the congenital nature of the chest wall defect. MH without a discrete hernia sac is rare, accounting for approximately 5% of all cases[14]. In this patient, the anteromedial diaphragmatic defect and absence of any prior operative manipulation in that region supported the diagnosis of a true MH, despite a history of multiple previous surgeries. No surgical scarring or synthetic material was identified at the site intraoperatively.

Given the diagnostic challenges and potential for serious complications, early recognition of rare hernia variants, such as TDIH, in combination with MH is essential. Tailored surgical repair remains the cornerstone of safe management.

## Conclusion

This case highlights the exceedingly rare coexistence of congenital MH and a TDIH secondary to an ipsilateral chest wall defect likely of congenital origin. Accurate diagnosis requires a high index of suspicion, particularly in patients with atypical presentations of the chest wall and diaphragmatic hernias. Tailored surgical approaches based on anatomical and clinical factors are essential as no universal repair strategy exists owing to the rarity of this condition.

## Data Availability

Not applicable.

## Research registration unique identifying number (UIN)

Not applicable for a single case report.
